# Bis{4-chloro-*N*′-[phen­yl(2-pyrid­yl)methyl­idene]benzohydrazidato-κ^2^ *N*′,*O*}cobalt(III) nitrate methanol disolvate

**DOI:** 10.1107/S1600536810032241

**Published:** 2010-08-18

**Authors:** Genhua Wu, Hui Ye, Dayu Wu

**Affiliations:** a Anhui Key Laboratory of Functional Coordination Compounds, School of Chemistry and Chemical Engineering, Anqing Teachers College, Anqing, 246011 Anhui, People’s Republic of China

## Abstract

In the title compound, [Co(C_19_H_13_ClN_3_O)_2_]NO_3_·2CH_3_OH, the central Co^III^ atom in the cation is surrounded by two tridentate ligands in a distorted octa­hedral fashion by four N and two O atoms. Classical O—H⋯O hydrogen bonds link both methanol solvent mol­ecules with the nitrate anion.

## Related literature

For related work on the mononuclear cobalt compound, see: Herchel & Boca (2005 ▶). For a dimetallic dicobalt(II) complex, see: Gavrilova *et al.* (2002 ▶). For a spin-crossover Fe^II^ complex, see: Wu *et al.* (2009 ▶).
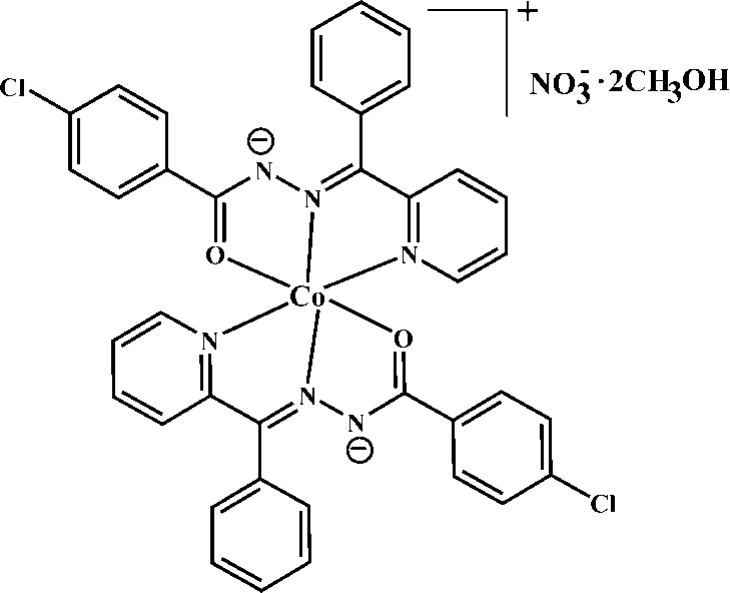


## Experimental

### 

#### Crystal data

[Co(C_19_H_13_ClN_3_O)_2_]NO_3_·2CH_4_O
*M* *_r_* = 854.57
Monoclinic, 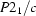

*a* = 12.914 (8) Å
*b* = 17.423 (11) Å
*c* = 17.451 (11) Å
β = 93.031 (8)°
*V* = 3921 (4) Å^3^
*Z* = 4
Mo *K*α radiation
μ = 0.63 mm^−1^
*T* = 293 K
0.31 × 0.23 × 0.21 mm

#### Data collection

Bruker SMART APEXII CCD area-detector diffractometer
Absorption correction: ψ scan (*SADABS*; Bruker, 1997 ▶) *T*_min_ = 0.839, *T*_max_ = 0.875
18168 measured reflections
6859 independent reflections
4829 reflections with *I* > 2σ(*I*)
*R*_int_ = 0.033

#### Refinement

*R*[*F* ^2^ > 2σ(*F* ^2^)] = 0.051
*wR*(*F* ^2^) = 0.156
*S* = 1.07
6859 reflections
516 parameters
H-atom parameters constrained
Δρ_max_ = 0.48 e Å^−3^
Δρ_min_ = −0.39 e Å^−3^

### 

Data collection: *SMART* (Bruker, 1997 ▶); cell refinement: *SAINT* (Bruker, 1997 ▶); data reduction: *SAINT*; program(s) used to solve structure: *SHELXS97* (Sheldrick, 2008 ▶); program(s) used to refine structure: *SHELXL97* (Sheldrick, 2008 ▶); molecular graphics: *SHELXTL* (Sheldrick, 2008 ▶); software used to prepare material for publication: *SHELXL97* and *PLATON* (Spek, 2009 ▶).

## Supplementary Material

Crystal structure: contains datablocks I, global. DOI: 10.1107/S1600536810032241/si2285sup1.cif

Structure factors: contains datablocks I. DOI: 10.1107/S1600536810032241/si2285Isup2.hkl

Additional supplementary materials:  crystallographic information; 3D view; checkCIF report

## Figures and Tables

**Table 1:** Selected bond lengths (Å)

Co1—N2	1.853 (3)
Co1—N5	1.859 (3)
Co1—O2	1.899 (2)
Co1—N4	1.909 (3)
Co1—O1	1.915 (2)
Co1—N1	1.921 (3)

**Table 2:** Hydrogen-bond geometry (Å, °)

*D*—H⋯*A*	*D*—H	H⋯*A*	*D*⋯*A*	*D*—H⋯*A*
O39—H39*D*⋯O3	0.82	1.94	2.747 (11)	167
O40—H40*D*⋯O3	0.85	2.16	2.873 (12)	142
O40—H40*D*⋯O5	0.85	2.20	2.963 (12)	150

**Fig. 1.**
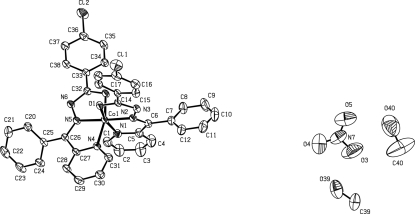
The molecular structure of the title compound, the thermal ellipsoids were drawn at 30% probability level.

**Fig. 2.**
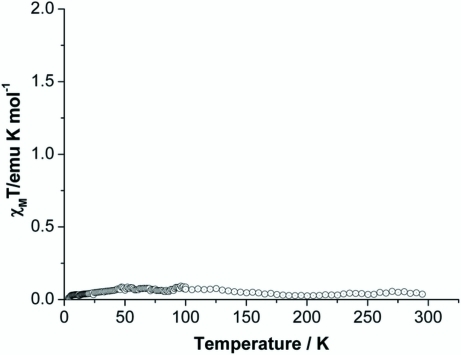
The temperature dependent curve χmT versus T for the title compound.

## supplementary crystallographic information

### Comment

The Co^III^ complex with the oxygen-containing Schiff-base ligand is important because of their ability to bind dioxygen. Among them, the most wanted targets include the artificial blood and respiratory systems. A novel aspect lies in the structural versatility of hexadentate Schiff-bases *versus*. imidazolidine complexes manifesting itself in a stabilization of various structural and optical isomers depending upon the chemical hardness of the metal centre (Herchel & Boca, 2005; Gavrilova *et al.*, 2002).

Our recent work indicated the *N*,*O*-donor tridentate ligand is suitable for the synthesis of spin-crossover materials (Wu *et al.*, 2009). One of the examples is reported by us, which interestingly showed the mixed-spin state and synergy between spin transition and magnetic interaction. Here, for the title compound, we used 2(*E*)-1-[(4-chlorophenyl)carbonyl]-2-[phenyl(pyridin-2-yl)methylidene] diazanide as ligand, a typical rigid tridentate donor to synthesize a mononuclear compound, and we report the crystal structure of the complex [Co(C_19_H_13_N_3_OCl)_2_]^+^(NO_3_)^-^(CH_3_OH)_2_ (Fig. 1). The coordination environments of Co^III^ ions are completed by two ligands with average Co—N bond length of 1.885 Å and Co—O 1.907 Å (Table 1). Classical hydrogen bonds O—H···O exist between both methanol solvent molecules and the nitrate anion with D···A distances between 2.747 (11) Å and 2.963 (12) Å (Table 2).

The temperature-dependent magnetic susceptibility was measured down to 1.8 K. In the χ.T *versus* T plot (Fig. 2), χ.T reaches a zero value within the whole temperature region, which is consistent with S = 0 ground state for cobalt(III).

### Experimental

A methanolic solution (25 ml) containing the ligand (0.2 mmol, 0.066 g) was added dropwise to Co(NO_3_)_2_.6H_2_O (0.1 mmol, 0.029 g). After stirring for 15 minutes, the dark solution was filtered. Red block-shaped crystals suitable for single-crystal X-ray diffraction were obtained by evaporating the resulting filtration in air for several days (yield: 56.2%). Anal calc (%). for C_40_H_34_Cl_2_CoN_7_O_7_: H 4.01 C 56.22 N 11.47. Found: H 4.12 C 56.32 N 11.21. The magnetic susceptibility χ was measured with a Quantum Design MPMS-5S SQUID magnetometer. Data were corrected for the diamagnetic contribution calculated from Pascal's constants.

### Refinement

C-bound H atoms were placed geometrically and allowed to ride during refinement with C—H = 0.93–0.96 Å with *U*_iso_(H) = 1.2 *U*_eq_(C). The hydroxy H atom of the methanol solvent molecule was located in a difference Fourier map and refined as riding with the parent atom with *U*_iso_(H) = 1.5*U*_eq_(O), O—H distances 0.82 and 0.85 Å.

### Crystal data

**Table:**

[Co(C_19_H_13_ClN_3_O)_2_]NO_3_·2CH_4_O	*F*(000) = 1760
*M**_r_* = 854.57	*D*_x_ = 1.448 Mg m^−^^3^
Monoclinic, *P*2_1_/*c*	Mo *K*α radiation, λ = 0.71073 Å
*a* = 12.914 (8) Å	Cell parameters from 6478 reflections
*b* = 17.423 (11) Å	θ = 2.0–29.8°
*c* = 17.451 (11) Å	µ = 0.63 mm^−^^1^
β = 93.031 (8)°	*T* = 293 K
*V* = 3921 (4) Å^3^	Block, dark-red
*Z* = 4	0.31 × 0.23 × 0.21 mm

### Data collection

**Table:**

Bruker SMART APEXII CCD area-detector diffractometer	6859 independent reflections
Radiation source: fine-focus sealed tube	4829 reflections with *I* > 2σ(*I*)
graphite	*R*_int_ = 0.033
φ and ω scans	θ_max_ = 25.0°, θ_min_ = 2.2°
Absorption correction: ψ scan (*SADABS*; Bruker, 1997)	*h* = −15→13
*T*_min_ = 0.839, *T*_max_ = 0.875	*k* = −20→20
18168 measured reflections	*l* = −20→16

### Refinement

**Table:**

Refinement on *F*^2^	Primary atom site location: structure-invariant direct methods
Least-squares matrix: full	Secondary atom site location: difference Fourier map
*R*[*F*^2^ > 2σ(*F*^2^)] = 0.051	Hydrogen site location: inferred from neighbouring sites
*wR*(*F*^2^) = 0.156	H-atom parameters constrained
*S* = 1.07	*w* = 1/[σ^2^(*F*_o_^2^) + (0.0912*P*)^2^] where *P* = (*F*_o_^2^ + 2*F*_c_^2^)/3
6859 reflections	(Δ/σ)_max_ < 0.001
516 parameters	Δρ_max_ = 0.48 e Å^−^^3^
0 restraints	Δρ_min_ = −0.39 e Å^−^^3^

### Special details

**Table:**

Experimental. The magnetic measurements were performed on Quantum Design SQUID, MPMS-5S magnetometer.
Geometry. All e.s.d.'s (except the e.s.d. in the dihedral angle between two l.s. planes) are estimated using the full covariance matrix. The cell e.s.d.'s are taken into account individually in the estimation of e.s.d.'s in distances, angles and torsion angles; correlations between e.s.d.'s in cell parameters are only used when they are defined by crystal symmetry. An approximate (isotropic) treatment of cell e.s.d.'s is used for estimating e.s.d.'s involving l.s. planes.
Refinement. Refinement of *F*^2^ against ALL reflections. The weighted *R*-factor *wR* and goodness of fit *S* are based on *F*^2^, conventional *R*-factors *R* are based on *F*, with *F* set to zero for negative *F*^2^. The threshold expression of *F*^2^ > σ(*F*^2^) is used only for calculating *R*-factors(gt) *etc*. and is not relevant to the choice of reflections for refinement. *R*-factors based on *F*^2^ are statistically about twice as large as those based on *F*, and *R*- factors based on ALL data will be even larger.

### Fractional atomic coordinates and isotropic or equivalent isotropic displacement parameters (Å^2^)

**Table:**

	*x*	*y*	*z*	*U*_iso_*/*U*_eq_	
Co1	0.78242 (3)	0.67158 (2)	0.45385 (2)	0.03739 (16)	
Cl1	1.25567 (8)	0.99362 (6)	0.58995 (7)	0.0794 (3)	
C1	0.5964 (3)	0.5811 (2)	0.4093 (2)	0.0584 (10)	
H1A	0.6374	0.5499	0.3801	0.070*	
Cl2	0.87476 (11)	0.29952 (6)	0.76381 (6)	0.0893 (4)	
C2	0.4924 (3)	0.5647 (3)	0.4130 (3)	0.0785 (13)	
H2A	0.4635	0.5232	0.3860	0.094*	
C3	0.4327 (3)	0.6090 (3)	0.4561 (3)	0.0953 (17)	
H3A	0.3628	0.5976	0.4598	0.143*	
C4	0.4764 (3)	0.6723 (2)	0.4953 (3)	0.0776 (13)	
H4A	0.4359	0.7038	0.5246	0.093*	
C5	0.5800 (3)	0.68681 (19)	0.4894 (2)	0.0499 (9)	
C6	0.6359 (2)	0.75209 (18)	0.52701 (18)	0.0428 (8)	
C7	0.5881 (2)	0.8070 (2)	0.5784 (2)	0.0494 (9)	
C8	0.5451 (3)	0.7802 (3)	0.6441 (2)	0.0721 (12)	
H8A	0.5458	0.7279	0.6551	0.087*	
C9	0.5006 (4)	0.8317 (3)	0.6939 (3)	0.0941 (16)	
H9A	0.4723	0.8140	0.7385	0.113*	
C10	0.4986 (4)	0.9092 (3)	0.6767 (4)	0.0972 (18)	
H10A	0.4676	0.9434	0.7093	0.117*	
C11	0.5417 (3)	0.9353 (3)	0.6127 (3)	0.0810 (13)	
H11A	0.5410	0.9876	0.6021	0.097*	
C12	0.5867 (3)	0.8852 (2)	0.5631 (2)	0.0618 (10)	
H12A	0.6161	0.9039	0.5193	0.074*	
C13	0.8957 (2)	0.78507 (16)	0.51482 (16)	0.0357 (7)	
C14	0.9853 (2)	0.83520 (16)	0.53516 (17)	0.0384 (7)	
C15	0.9705 (3)	0.90836 (18)	0.5657 (2)	0.0473 (8)	
H15A	0.9037	0.9249	0.5745	0.057*	
C16	1.0538 (3)	0.95642 (19)	0.5829 (2)	0.0541 (9)	
H16A	1.0436	1.0049	0.6035	0.065*	
C17	1.1516 (3)	0.9312 (2)	0.5690 (2)	0.0532 (9)	
C18	1.1684 (3)	0.8602 (2)	0.5394 (2)	0.0556 (9)	
H18A	1.2355	0.8447	0.5301	0.067*	
C19	1.0863 (3)	0.8113 (2)	0.5233 (2)	0.0501 (9)	
H19A	1.0982	0.7624	0.5044	0.060*	
C20	0.9368 (3)	0.47364 (19)	0.2934 (2)	0.0512 (9)	
H20A	0.9804	0.4804	0.3370	0.061*	
C21	0.9521 (3)	0.4125 (2)	0.2450 (3)	0.0675 (11)	
H21A	1.0063	0.3784	0.2560	0.081*	
C22	0.8877 (4)	0.4018 (2)	0.1807 (2)	0.0760 (13)	
H22A	0.8986	0.3605	0.1483	0.091*	
C23	0.8071 (4)	0.4517 (2)	0.1639 (2)	0.0698 (12)	
H23A	0.7630	0.4439	0.1207	0.084*	
C24	0.7922 (3)	0.5131 (2)	0.2112 (2)	0.0541 (9)	
H24A	0.7385	0.5474	0.1994	0.065*	
C25	0.8568 (2)	0.52489 (18)	0.27717 (17)	0.0408 (8)	
C26	0.8359 (2)	0.59022 (17)	0.32795 (17)	0.0391 (7)	
C27	0.8125 (2)	0.66884 (17)	0.29955 (17)	0.0406 (7)	
C28	0.8283 (3)	0.69599 (19)	0.22675 (19)	0.0501 (9)	
H28A	0.8529	0.6634	0.1896	0.060*	
C29	0.8071 (3)	0.7723 (2)	0.2094 (2)	0.0594 (10)	
H29A	0.8168	0.7909	0.1604	0.071*	
C30	0.7716 (3)	0.8203 (2)	0.2648 (2)	0.0594 (10)	
H30A	0.7573	0.8715	0.2537	0.071*	
C31	0.7577 (3)	0.79147 (19)	0.3368 (2)	0.0526 (9)	
H31A	0.7337	0.8238	0.3744	0.063*	
C32	0.8300 (2)	0.53820 (17)	0.51756 (17)	0.0386 (7)	
C33	0.8387 (2)	0.47777 (17)	0.57681 (17)	0.0373 (7)	
C34	0.7889 (2)	0.48666 (18)	0.64438 (19)	0.0435 (8)	
H34A	0.7487	0.5301	0.6514	0.052*	
C35	0.7981 (3)	0.43203 (19)	0.7017 (2)	0.0512 (9)	
H35A	0.7636	0.4383	0.7467	0.061*	
C36	0.8591 (3)	0.36758 (19)	0.69139 (19)	0.0516 (9)	
C37	0.9087 (3)	0.35630 (19)	0.6240 (2)	0.0504 (9)	
H37A	0.9486	0.3126	0.6172	0.061*	
C38	0.8977 (3)	0.41166 (17)	0.56659 (19)	0.0428 (8)	
H38A	0.9301	0.4045	0.5208	0.051*	
N1	0.6394 (2)	0.64084 (15)	0.44688 (15)	0.0445 (7)	
N2	0.73280 (19)	0.75294 (13)	0.50965 (14)	0.0372 (6)	
N3	0.80414 (19)	0.80719 (14)	0.53669 (14)	0.0390 (6)	
N4	0.7780 (2)	0.71727 (15)	0.35430 (15)	0.0423 (6)	
N5	0.83192 (19)	0.58678 (14)	0.40247 (14)	0.0388 (6)	
N6	0.8493 (2)	0.52095 (14)	0.44585 (14)	0.0406 (6)	
O1	0.91181 (16)	0.72240 (11)	0.47687 (12)	0.0416 (5)	
O2	0.80192 (16)	0.60633 (11)	0.54059 (11)	0.0412 (5)	
O39	0.3498 (5)	1.5978 (3)	0.6311 (3)	0.165 (2)	
H39D	0.3843	1.6034	0.6715	0.248*	
C39	0.2696 (5)	1.6509 (3)	0.6272 (4)	0.125 (2)	
H39A	0.2975	1.7016	0.6341	0.187*	
H39B	0.2228	1.6403	0.6669	0.187*	
H39C	0.2329	1.6474	0.5781	0.187*	
C40	0.3173 (11)	1.7336 (7)	0.8722 (10)	0.362 (12)	
H40A	0.3084	1.7797	0.9011	0.543*	
H40B	0.2507	1.7119	0.8580	0.543*	
H40C	0.3529	1.7453	0.8267	0.543*	
O40	0.3808 (7)	1.6761 (6)	0.9207 (6)	0.279 (4)	
H40D	0.4158	1.6491	0.8909	0.419*	
O3	0.4443 (5)	1.6345 (6)	0.7710 (5)	0.279 (5)	
O4	0.5735 (5)	1.5770 (3)	0.7264 (4)	0.179 (3)	
O5	0.5666 (5)	1.6126 (4)	0.8508 (4)	0.188 (3)	
N7	0.5329 (5)	1.6068 (4)	0.7830 (5)	0.130 (2)	

### Atomic displacement parameters (Å^2^)

**Table:**

	*U*^11^	*U*^22^	*U*^33^	*U*^12^	*U*^13^	*U*^23^
Co1	0.0474 (3)	0.0359 (3)	0.0290 (3)	0.00039 (18)	0.00347 (18)	−0.00440 (17)
Cl1	0.0703 (7)	0.0821 (7)	0.0852 (8)	−0.0326 (6)	−0.0027 (6)	0.0005 (6)
C1	0.062 (2)	0.057 (2)	0.056 (2)	−0.0065 (18)	0.0029 (19)	−0.0148 (18)
Cl2	0.1574 (12)	0.0600 (6)	0.0515 (7)	0.0127 (7)	0.0158 (7)	0.0175 (5)
C2	0.062 (3)	0.081 (3)	0.093 (3)	−0.022 (2)	0.004 (2)	−0.033 (3)
C3	0.058 (3)	0.106 (4)	0.124 (5)	−0.025 (3)	0.017 (3)	−0.042 (3)
C4	0.054 (2)	0.084 (3)	0.096 (4)	−0.012 (2)	0.014 (2)	−0.029 (3)
C5	0.049 (2)	0.051 (2)	0.050 (2)	−0.0032 (16)	0.0047 (16)	−0.0083 (16)
C6	0.0439 (19)	0.0461 (19)	0.0386 (19)	0.0027 (15)	0.0043 (15)	−0.0054 (14)
C7	0.0401 (19)	0.058 (2)	0.051 (2)	0.0024 (16)	0.0030 (16)	−0.0157 (17)
C8	0.071 (3)	0.075 (3)	0.072 (3)	0.006 (2)	0.030 (2)	−0.006 (2)
C9	0.088 (4)	0.126 (5)	0.072 (3)	0.002 (3)	0.037 (3)	−0.025 (3)
C10	0.072 (3)	0.103 (4)	0.118 (5)	0.013 (3)	0.016 (3)	−0.055 (4)
C11	0.072 (3)	0.066 (3)	0.105 (4)	0.012 (2)	0.007 (3)	−0.025 (3)
C12	0.059 (2)	0.057 (2)	0.070 (3)	0.0083 (18)	0.008 (2)	−0.009 (2)
C13	0.0474 (19)	0.0366 (17)	0.0238 (16)	0.0003 (14)	0.0072 (14)	−0.0009 (13)
C14	0.0463 (18)	0.0422 (18)	0.0275 (16)	0.0000 (14)	0.0082 (14)	−0.0010 (13)
C15	0.051 (2)	0.0429 (19)	0.048 (2)	−0.0008 (15)	0.0092 (16)	−0.0030 (15)
C16	0.067 (2)	0.0420 (19)	0.053 (2)	−0.0088 (17)	0.0028 (19)	−0.0034 (16)
C17	0.058 (2)	0.060 (2)	0.043 (2)	−0.0198 (18)	0.0044 (17)	0.0017 (17)
C18	0.044 (2)	0.070 (2)	0.054 (2)	−0.0043 (18)	0.0075 (17)	−0.0018 (19)
C19	0.052 (2)	0.055 (2)	0.044 (2)	0.0008 (16)	0.0057 (16)	−0.0092 (16)
C20	0.056 (2)	0.050 (2)	0.047 (2)	0.0003 (17)	0.0032 (17)	−0.0118 (16)
C21	0.078 (3)	0.054 (2)	0.072 (3)	0.0069 (19)	0.019 (2)	−0.014 (2)
C22	0.123 (4)	0.051 (2)	0.055 (3)	−0.014 (3)	0.021 (3)	−0.023 (2)
C23	0.106 (3)	0.064 (3)	0.039 (2)	−0.016 (2)	−0.002 (2)	−0.0195 (19)
C24	0.072 (2)	0.052 (2)	0.038 (2)	−0.0066 (18)	−0.0035 (17)	−0.0052 (16)
C25	0.052 (2)	0.0423 (18)	0.0292 (17)	−0.0092 (15)	0.0079 (15)	−0.0073 (13)
C26	0.0453 (18)	0.0419 (18)	0.0302 (18)	−0.0010 (14)	0.0011 (14)	−0.0064 (13)
C27	0.0507 (19)	0.0435 (18)	0.0271 (17)	−0.0018 (14)	−0.0014 (14)	−0.0068 (13)
C28	0.072 (2)	0.048 (2)	0.0307 (19)	−0.0016 (17)	0.0037 (16)	−0.0064 (15)
C29	0.088 (3)	0.055 (2)	0.035 (2)	−0.0024 (19)	−0.0003 (19)	0.0044 (17)
C30	0.084 (3)	0.046 (2)	0.047 (2)	0.0085 (18)	0.000 (2)	0.0079 (17)
C31	0.073 (2)	0.045 (2)	0.041 (2)	0.0108 (17)	0.0037 (17)	−0.0017 (16)
C32	0.0461 (18)	0.0359 (17)	0.0339 (18)	−0.0040 (14)	0.0016 (14)	−0.0049 (13)
C33	0.0436 (18)	0.0383 (17)	0.0301 (17)	−0.0037 (13)	0.0015 (14)	−0.0062 (13)
C34	0.051 (2)	0.0398 (18)	0.0396 (19)	0.0005 (14)	0.0045 (15)	−0.0051 (14)
C35	0.068 (2)	0.051 (2)	0.036 (2)	−0.0028 (17)	0.0147 (17)	−0.0049 (16)
C36	0.078 (2)	0.0405 (19)	0.036 (2)	−0.0068 (17)	0.0023 (18)	0.0011 (15)
C37	0.067 (2)	0.0368 (18)	0.047 (2)	0.0045 (16)	0.0025 (18)	−0.0014 (15)
C38	0.054 (2)	0.0405 (18)	0.0342 (18)	−0.0029 (15)	0.0056 (15)	−0.0042 (14)
N1	0.0515 (16)	0.0400 (15)	0.0417 (16)	−0.0025 (12)	−0.0008 (13)	−0.0061 (12)
N2	0.0439 (16)	0.0364 (14)	0.0312 (15)	−0.0005 (11)	0.0014 (11)	−0.0027 (11)
N3	0.0439 (16)	0.0395 (14)	0.0340 (15)	−0.0017 (12)	0.0055 (12)	−0.0058 (11)
N4	0.0499 (16)	0.0430 (16)	0.0338 (15)	0.0029 (12)	0.0010 (12)	−0.0015 (12)
N5	0.0501 (16)	0.0365 (14)	0.0297 (15)	−0.0007 (11)	0.0018 (12)	−0.0017 (11)
N6	0.0547 (17)	0.0359 (14)	0.0313 (15)	0.0005 (12)	0.0023 (12)	−0.0033 (11)
O1	0.0467 (12)	0.0415 (12)	0.0370 (13)	0.0015 (9)	0.0051 (10)	−0.0085 (10)
O2	0.0580 (13)	0.0369 (12)	0.0291 (12)	0.0029 (10)	0.0052 (10)	−0.0041 (9)
O39	0.203 (5)	0.130 (4)	0.168 (5)	−0.012 (4)	0.056 (4)	−0.046 (3)
C39	0.120 (5)	0.086 (4)	0.173 (7)	0.009 (4)	0.054 (4)	−0.013 (4)
C40	0.288 (16)	0.233 (14)	0.54 (3)	0.011 (11)	−0.218 (17)	0.054 (16)
O40	0.248 (9)	0.326 (11)	0.263 (10)	−0.029 (7)	0.013 (7)	−0.119 (8)
O3	0.135 (5)	0.431 (12)	0.269 (9)	0.069 (6)	0.003 (5)	0.133 (9)
O4	0.156 (5)	0.140 (4)	0.245 (7)	−0.034 (3)	0.060 (5)	−0.033 (4)
O5	0.145 (5)	0.209 (6)	0.206 (7)	−0.063 (4)	−0.034 (4)	0.095 (5)
N7	0.088 (4)	0.115 (5)	0.188 (8)	−0.012 (3)	0.000 (5)	0.069 (5)

### Geometric parameters (Å, °)

**Table:**

Co1—N2	1.853 (3)	C21—H21A	0.9300
Co1—N5	1.859 (3)	C22—C23	1.377 (6)
Co1—O2	1.899 (2)	C22—H22A	0.9300
Co1—N4	1.909 (3)	C23—C24	1.371 (5)
Co1—O1	1.915 (2)	C23—H23A	0.9300
Co1—N1	1.921 (3)	C24—C25	1.400 (5)
Cl1—C17	1.752 (3)	C24—H24A	0.9300
C1—N1	1.336 (4)	C25—C26	1.476 (4)
C1—C2	1.378 (5)	C26—N5	1.306 (4)
C1—H1A	0.9300	C26—C27	1.482 (4)
Cl2—C36	1.737 (3)	C27—N4	1.367 (4)
C2—C3	1.347 (6)	C27—C28	1.381 (5)
C2—H2A	0.9300	C28—C29	1.387 (5)
C3—C4	1.400 (6)	C28—H28A	0.9300
C3—H3A	0.9300	C29—C30	1.375 (5)
C4—C5	1.371 (5)	C29—H29A	0.9300
C4—H4A	0.9300	C30—C31	1.374 (5)
C5—N1	1.357 (4)	C30—H30A	0.9300
C5—C6	1.481 (4)	C31—N4	1.351 (4)
C6—N2	1.304 (4)	C31—H31A	0.9300
C6—C7	1.469 (4)	C32—O2	1.310 (3)
C7—C8	1.381 (5)	C32—N6	1.324 (4)
C7—C12	1.390 (5)	C32—C33	1.476 (4)
C8—C9	1.394 (6)	C33—C34	1.381 (4)
C8—H8A	0.9300	C33—C38	1.398 (4)
C9—C10	1.382 (7)	C34—C35	1.381 (5)
C9—H9A	0.9300	C34—H34A	0.9300
C10—C11	1.353 (7)	C35—C36	1.389 (5)
C10—H10A	0.9300	C35—H35A	0.9300
C11—C12	1.378 (5)	C36—C37	1.382 (5)
C11—H11A	0.9300	C37—C38	1.393 (4)
C12—H12A	0.9300	C37—H37A	0.9300
C13—O1	1.299 (3)	C38—H38A	0.9300
C13—N3	1.320 (4)	N2—N3	1.385 (3)
C13—C14	1.478 (4)	N5—N6	1.386 (3)
C14—C19	1.395 (5)	O39—C39	1.387 (7)
C14—C15	1.398 (4)	O39—H39D	0.8200
C15—C16	1.383 (5)	C39—H39A	0.9600
C15—H15A	0.9300	C39—H39B	0.9600
C16—C17	1.371 (5)	C39—H39C	0.9600
C16—H16A	0.9300	C40—O40	1.523 (12)
C17—C18	1.362 (5)	C40—H40A	0.9600
C18—C19	1.378 (5)	C40—H40B	0.9600
C18—H18A	0.9300	C40—H40C	0.9600
C19—H19A	0.9300	O40—H40D	0.8500
C20—C21	1.380 (5)	O3—N7	1.249 (7)
C20—C25	1.383 (5)	O4—N7	1.256 (8)
C20—H20A	0.9300	O5—N7	1.243 (8)
C21—C22	1.373 (6)		
			
N2—Co1—N5	176.99 (11)	C24—C23—C22	119.5 (4)
N2—Co1—O2	94.25 (11)	C24—C23—H23A	120.2
N5—Co1—O2	82.78 (10)	C22—C23—H23A	120.2
N2—Co1—N4	99.48 (11)	C23—C24—C25	120.9 (4)
N5—Co1—N4	83.51 (11)	C23—C24—H24A	119.5
O2—Co1—N4	166.22 (10)	C25—C24—H24A	119.5
N2—Co1—O1	81.85 (10)	C20—C25—C24	118.6 (3)
N5—Co1—O1	98.64 (10)	C20—C25—C26	122.2 (3)
O2—Co1—O1	91.84 (9)	C24—C25—C26	119.1 (3)
N4—Co1—O1	88.96 (10)	N5—C26—C25	125.5 (3)
N2—Co1—N1	83.59 (11)	N5—C26—C27	111.0 (3)
N5—Co1—N1	95.86 (11)	C25—C26—C27	123.5 (3)
O2—Co1—N1	88.29 (10)	N4—C27—C28	120.1 (3)
N4—Co1—N1	94.38 (11)	N4—C27—C26	113.9 (3)
O1—Co1—N1	165.41 (10)	C28—C27—C26	126.0 (3)
N1—C1—C2	121.3 (3)	C27—C28—C29	119.5 (3)
N1—C1—H1A	119.4	C27—C28—H28A	120.3
C2—C1—H1A	119.4	C29—C28—H28A	120.3
C3—C2—C1	119.6 (4)	C30—C29—C28	119.9 (3)
C3—C2—H2A	120.2	C30—C29—H29A	120.0
C1—C2—H2A	120.2	C28—C29—H29A	120.0
C2—C3—C4	119.8 (4)	C31—C30—C29	119.0 (3)
C2—C3—H3A	120.1	C31—C30—H30A	120.5
C4—C3—H3A	120.1	C29—C30—H30A	120.5
C5—C4—C3	118.6 (4)	N4—C31—C30	121.5 (3)
C5—C4—H4A	120.7	N4—C31—H31A	119.2
C3—C4—H4A	120.7	C30—C31—H31A	119.2
N1—C5—C4	120.9 (3)	O2—C32—N6	124.4 (3)
N1—C5—C6	114.8 (3)	O2—C32—C33	116.3 (3)
C4—C5—C6	124.3 (3)	N6—C32—C33	119.3 (3)
N2—C6—C7	125.0 (3)	C34—C33—C38	118.9 (3)
N2—C6—C5	110.9 (3)	C34—C33—C32	119.8 (3)
C7—C6—C5	124.0 (3)	C38—C33—C32	121.4 (3)
C8—C7—C12	119.3 (3)	C35—C34—C33	121.0 (3)
C8—C7—C6	119.2 (3)	C35—C34—H34A	119.5
C12—C7—C6	121.5 (3)	C33—C34—H34A	119.5
C7—C8—C9	119.7 (4)	C34—C35—C36	119.4 (3)
C7—C8—H8A	120.1	C34—C35—H35A	120.3
C9—C8—H8A	120.1	C36—C35—H35A	120.3
C10—C9—C8	119.9 (5)	C37—C36—C35	121.1 (3)
C10—C9—H9A	120.1	C37—C36—Cl2	118.7 (3)
C8—C9—H9A	120.1	C35—C36—Cl2	120.2 (3)
C11—C10—C9	120.2 (4)	C36—C37—C38	118.6 (3)
C11—C10—H10A	119.9	C36—C37—H37A	120.7
C9—C10—H10A	119.9	C38—C37—H37A	120.7
C10—C11—C12	120.7 (4)	C37—C38—C33	121.0 (3)
C10—C11—H11A	119.6	C37—C38—H38A	119.5
C12—C11—H11A	119.6	C33—C38—H38A	119.5
C11—C12—C7	120.1 (4)	C1—N1—C5	119.8 (3)
C11—C12—H12A	119.9	C1—N1—Co1	128.4 (2)
C7—C12—H12A	119.9	C5—N1—Co1	111.7 (2)
O1—C13—N3	124.2 (3)	C6—N2—N3	123.9 (2)
O1—C13—C14	118.4 (3)	C6—N2—Co1	118.4 (2)
N3—C13—C14	117.4 (3)	N3—N2—Co1	117.31 (19)
C19—C14—C15	118.4 (3)	C13—N3—N2	107.0 (2)
C19—C14—C13	120.9 (3)	C31—N4—C27	120.0 (3)
C15—C14—C13	120.6 (3)	C31—N4—Co1	126.9 (2)
C16—C15—C14	121.0 (3)	C27—N4—Co1	112.5 (2)
C16—C15—H15A	119.5	C26—N5—N6	124.6 (3)
C14—C15—H15A	119.5	C26—N5—Co1	118.5 (2)
C17—C16—C15	118.7 (3)	N6—N5—Co1	116.38 (19)
C17—C16—H16A	120.6	C32—N6—N5	107.2 (2)
C15—C16—H16A	120.6	C13—O1—Co1	109.54 (19)
C18—C17—C16	121.6 (3)	C32—O2—Co1	108.96 (18)
C18—C17—Cl1	120.5 (3)	C39—O39—H39D	109.5
C16—C17—Cl1	117.9 (3)	O39—C39—H39A	109.5
C17—C18—C19	120.2 (3)	O39—C39—H39B	109.5
C17—C18—H18A	119.9	H39A—C39—H39B	109.5
C19—C18—H18A	119.9	O39—C39—H39C	109.5
C18—C19—C14	120.0 (3)	H39A—C39—H39C	109.5
C18—C19—H19A	120.0	H39B—C39—H39C	109.5
C14—C19—H19A	120.0	O40—C40—H40A	109.5
C21—C20—C25	120.2 (3)	O40—C40—H40B	109.5
C21—C20—H20A	119.9	H40A—C40—H40B	109.5
C25—C20—H20A	119.9	O40—C40—H40C	109.5
C22—C21—C20	120.3 (4)	H40A—C40—H40C	109.5
C22—C21—H21A	119.9	H40B—C40—H40C	109.5
C20—C21—H21A	119.9	C40—O40—H40D	108.1
C21—C22—C23	120.5 (4)	O5—N7—O3	113.6 (9)
C21—C22—H22A	119.8	O5—N7—O4	130.0 (8)
C23—C22—H22A	119.8	O3—N7—O4	116.4 (9)

### Hydrogen-bond geometry (Å, °)

**Table:**

*D*—H···*A*	*D*—H	H···*A*	*D*···*A*	*D*—H···*A*
O39—H39D···O3	0.82	1.94	2.747 (11)	167.
O40—H40D···O3	0.85	2.16	2.873 (12)	142.
O40—H40D···O5	0.85	2.20	2.963 (12)	150.
